# The status and hotspot analysis of research on extracellular vesicles and osteoarthritis: a bibliometric analysis

**DOI:** 10.3389/fphar.2025.1484437

**Published:** 2025-03-31

**Authors:** Wen Hao Zhang, Wen Yuan Xiang, Lin Yi, Rui Fang

**Affiliations:** ^1^ The Fourth Clinical College of Xinjiang Medical University, Urumqi, China; ^2^ Department of Orthopaedic, Institute of Traditional Chinese Medicine Hospital of Xinjiang Uygur Autonomous Region, Urumqi, China; ^3^ Department of Orthopaedic, Xinjiang Uygur Autonomous Region Institute of Traditional Chinese Medicine, Urumqi, China; ^4^ Department of orthopaedic, The Fourth Affiliated Hospital of Xinjiang Medical University, Urumqi, China

**Keywords:** osteoarthritis, extracellular vesicles, bibliometric, hotspots, mesenchymal stem cell

## Abstract

**Background:**

Degenerative joint disease, known as osteoarthritis (OA), is characterized by pain, swelling, and decreased mobility. The illness has a major negative influence on patients’ quality of life and is common around the world, especially among older people. Nevertheless, there are insufficient possibilities for early diagnosis and therapy. Extracellular vesicles, or EVs, control the immune response, tissue healing, and cellular communication.

**Methods:**

This work offers a bibliometric representation of the areas of focus and correlations between extracellular vesicles and osteoarthritis. We searched for osteoarthritis and extracellular vesicles in publications in the Web of Science Core Collection (WoSCC) database. Bibliometrics, an R package, CiteSpace 6.1. R2, and VOSviewer 1.6.17 were used to perform bibliometric analyses of concentration fields, trends, and relevant factors.

**Results:**

944 papers from 59 nations were published; the countries that contributed the most to the field were China, the USA, and Italy. Professors Laura and Enrico are the top contributors. Sichuan University, Istituto Ortopedico Galeazzi, and Shanghai Jiao Tong University are the top three universities. The International Journal of Molecular Sciences is an excellent publication. Exosome, expression, knee osteoarthritis, extracellular vesicle, mesenchymal stem cell, osteoarthritis, and inflammation are the most often occurring keywords.

**Conclusion:**

These results suggest areas of interest and focus for future research on EVs and OA. This trend suggests that the volume of literature on OA and EVs will continue to rise, with more research being published in the future. This study helps scholars understand current research hotspots in the field and may inspire future research.

## 1 Introduction

Osteoarthritis OA is a degenerative disease of the bones and joints that can be recognized by inflammation of the synovium, secondary osteophytes, and loss of articular cartilage. The most common clinical manifestations include stiffness, discomfort, edema, and dysfunction (Hunter, 2015; Sharma, 2021). With an approximate global burden of 16%, the prevalence of OA has dramatically increased due to the growing number of elderly and obese people (Cui et al., 2020). In China, the prevalence of OA exceeds 50% in people with knee pain over the age of 65, and the prevalence of OA exceeds 80% in those over the age of 75 with knee pain (Tang et al., 2016).

OA has a long disease progression period and is strongly associated with older age, gender, overweight, residential environment, and genetic predisposition (Di et al., 2024; Georgiev and Angelov, 2019; Simão et al., 2019). It also has a lengthy progression time. OA may result in decreased quality of life, disability, loss of joint function, and higher medical costs for patients (Ali et al., 2017). Patients with KOA are often assessed using the Kellgren-Lawrence (K-L) classification. Total knee arthroplasty (TKA) and unicompartmental knee arthroplasty (UKA) are frequently utilized for patients with grade III or IV K-L classification (Mancuso et al., 2016; Yao et al., 2023; Zhao et al., 2022).

However, there are issues related to high surgical costs, infections, blood embolism, implant lifespan, and longevity (Gililland et al., 2012; Sloan and Lee, 2021). Patients with early OA, such as those classified as grade I or II in the K-L grading system, are frequently treated with PRP (platelet-rich plasma), sodium hyaluronate, and non-steroidal anti-inflammatory medicines (NSAIDs) (Feng et al., 2023; Sánchez et al., 2021; Su et al., 2018). However, most of these treatments only moderate its symptoms of OA and do not fundamentally reverse its pathological changes. Therefore, exploring treatment modalities for OA is a worthwhile topic for in-depth investigation.

Stem cell therapy has become available as an experimental and clinical treatment option for adolescent OA. However, there are risks, such as safety and potential tumour differentiation (Damjanov and Andrews, 2016). EVs, a type of cell-free therapy, have been extensively studied for their advantages in regeneration, immunomodulation, and inflammation modulation by researchers (Liu et al., 2023; Lv et al., 2020). EVs are membrane structures that frequently originate from cells and have a diameter that ranges from 100 nm to 1 µm. Initially thought to be primarily metabolic waste products by researchers, EVs now represent a broad category of substances (Vandergriff et al., 2018; Andaloussi et al., 2013). All prokaryotic and eukaryotic cells studied to date produce and release phospholipid bilayer biovesicles known as EVs, containing abundant lipids, protein, ribonucleic acid, and other physiologically active substances. EVs are typically classified based on their vesicle diameter into apoptotic bodies, microvesicles, microparticles (Bheri et al., 2020; Andaloussi et al., 2013), and nano-vesicles (Zhang et al., 2021; Pang et al., 2023).

Apoptotic bodies are vesicles released by apoptotic cells containing intracellular proteins and nucleic acids (Xu et al., 2019). Microvesicles are small membranous vesicles secreted by microorganisms, containing proteins and lipids from the microorganism’s surface (Wang X. et al., 2022). Small vesicles with membranes called microvesicles can get into cells and deliver lipids, proteins, and genetic information (Xie et al., 2016). EVs have shown promising results in treating conditions such as myocardial infarction (Nian and Fu, 2023), spinal cord injury (Fan et al., 2022), tumors (Shao et al., 2022), diabetes (Sun et al., 2021), and others in previous studies.

Bibliometrics is an essential tool for research assessment, systematically analyzing literature to provide researchers with comprehensive insights into data and trends (Zhang et al., 2023a; Zhang et al., 2023b). It provides a comprehensive overview of research trends, hotspots, and developmental behaviors within a particular field or matter. Bibliometrics helps assess the quantity and quality of publications on current topics, institutions, and regions. It also forecasts future research directions and guides research trends (Zhou et al., 2023; Hu et al., 2023). While studies on EVs and OA are present in existing bibliographic analyses, fewer studies have focused on bibliometric analyses specifically.

Using the WoSCC database, bibliometric tools like CiteSpace and VOSviewer, and websites like bibliometric.com, this study provides a bibliometric analysis of documents about EVs and OA. The analysis covers aspects such as country, institution, authors, journals, highly cited publications, and keywords. The study sheds light on hotspots, research trends, and upcoming advancements in EVs and OA.

## 2 Materials and methods

### 2.1 Data collection

**FIGURE 1:**
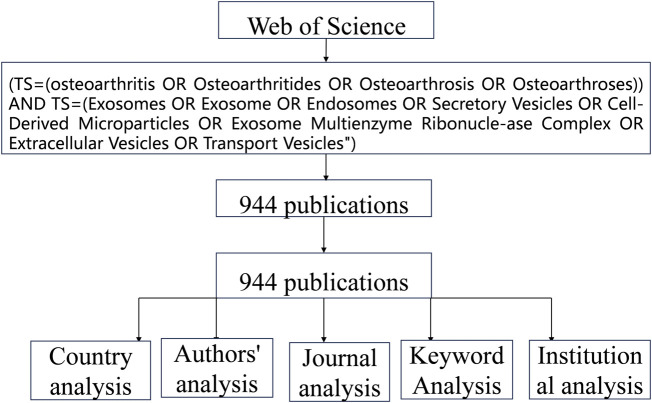
Flow diagram of the include articles.

This study employed the Web of Science Core Collection (WoSCC), a globally indexed scientific database. To ensure data accuracy, searches were conducted, data downloaded, and data analysis performed on 1 January 2025. The search covered publications from 1 January 2000 to 31 December 2024, with the following Boolean query: *TS=(Osteoarthritis OR Osteoarthritides OR Osteoarthrosis OR Osteoarthroses) AND TS=(Exosomes OR Extracellular Vesicles OR Secretory Vesicles OR Cell-Derived Microparticles)*. The search adhered strictly to the predefined keywords, and retrieved records underwent systematic analysis. Full-record datasets, including abstracts and citations, were exported and processed as outlined in Figure 1 (data extraction workflow).

## 3 Results

### 3.1 Trend analysis of publications

**FIGURE 2:**
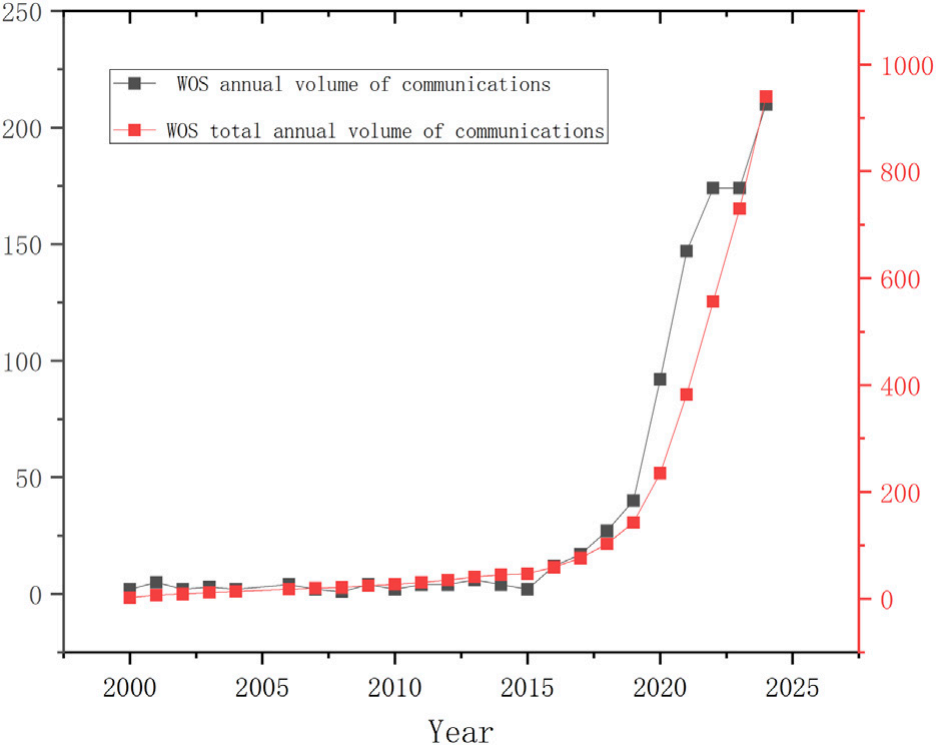
A Treands in total annual number of publications related to EVs and OA publications.

By conducting a keyword-based search in the Web of Science Core Collection (WoSCC) database, a total of 944 research articles related to extracellular vesicles (EVs) and osteoarthritis (OA) were identified. The first study exploring the relationship between EVs and OA dates back to 2000. From 2000 to 2015, the number of publications in this field remained relatively low (as shown in Figure 2), indicating limited academic attention during this period. Starting in 2016, the annual number of publications surpassed double digits for the first time, with 12 papers published that year.

Between 2016 and 2019, the number of publications continued to grow, reflecting a significant increase in research output. In 2021, the number of publications exceeded 100 (reaching 147), and by 2024, it peaked at 210. Although there was a slight decline in the number of publications in 2023 compared to 2022, the overall trend remained highly productive. As illustrated in Figure 2, research on EVs in OA has shown a consistent upward trajectory over the past 5 years, gradually emerging as an important and rapidly growing field. This trend is further supported by the increasing proportion of original research articles compared to review articles, highlighting the growing focus on the therapeutic, diagnostic, and mechanistic roles of EVs in OA. The substantial and valuable research output in this field underscores its potential for further exploration and development.

### 3.2 Countries/regions analysis

A visual analysis of the 944 publications from 2000 to 2024, focusing on the countries and regions involved, was conducted using VOSviewer software. When the threshold was set to one publication, 59 countries met this criterion; when the threshold was increased to four publications, 32 countries qualified.

**FIGURE 3:**
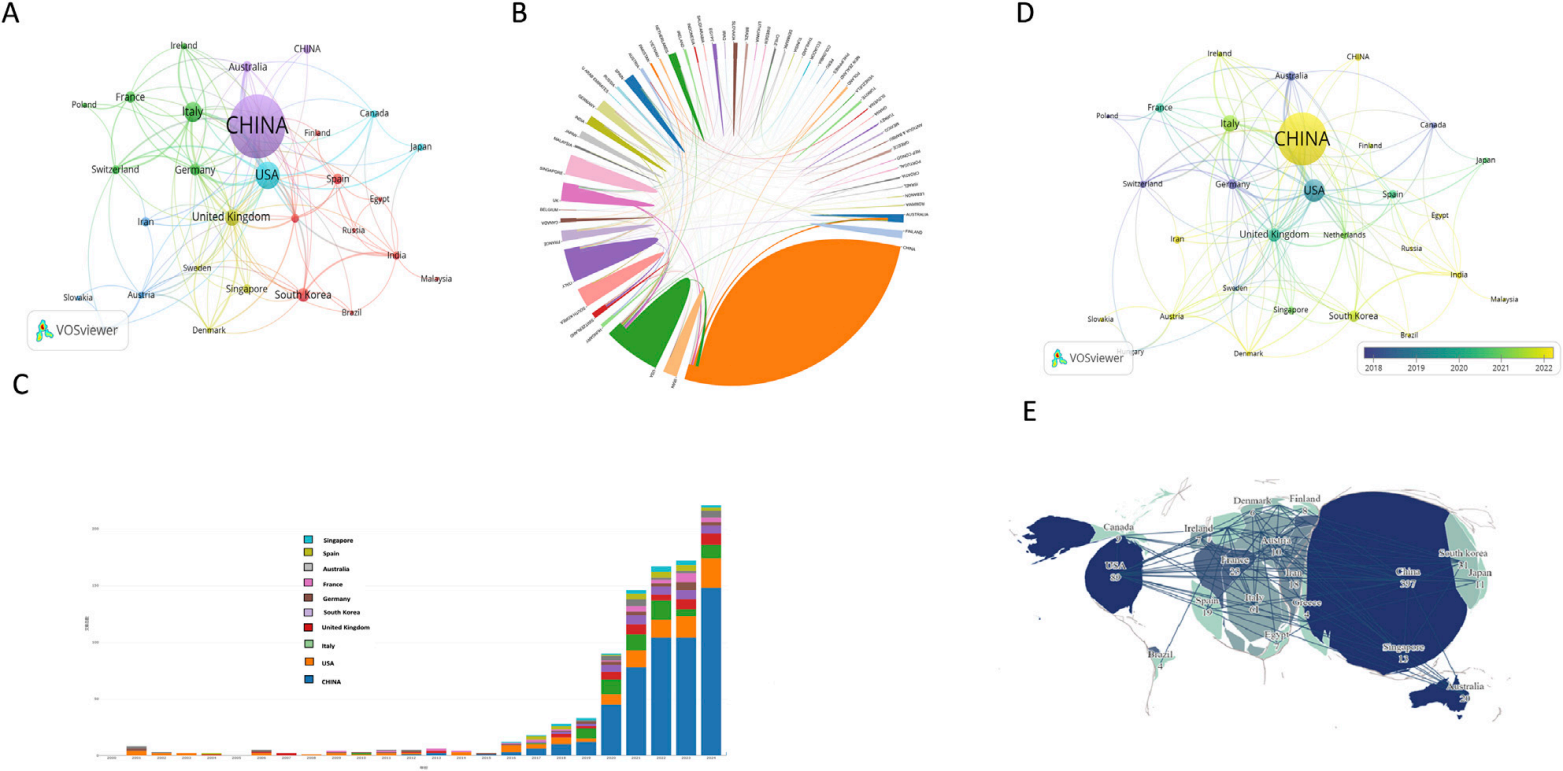
National collaborative analysis of extracellular vesicles and osteoarthritis **(A)** Collaboration between countries or regions based on VOSviewer; **(B)** The coauthorship network map of countries; **(C)** Annual number of publications in different countries **(D)** Map of the network of countries working together in time; **(E)** The coauthorship network map of countries.

As shown in Figure 3A, five clusters were identified, with the largest cluster comprising China and the United States. This cluster includes China, the United States, Japan, and Australia, encompassing 683 articles, which account for 72.35% of the total publications (Table 1). China leads with 518 articles, representing 54.87% of the total, and has garnered 15,077 citations, with an average of 29.11 citations per article and an H-index of 52. The total link strength of 82 highlights China’s prominent position in this research field and its extensive international collaborations, as illustrated in Figures 3B, C.

**TABLE 1:** Top 10 countries in terms of the number of published papers.

Rank	Country	Record	Citations	Total link strength	Average citations	H-index
1	China	518	15077	82	29.11	52
2	United States of America	124	5,838	112	47.08	30
3	Italy	73	1741	53	23.85	23
4	United Kingdom	52	901	67	17.33	14
5	South Korea	41	924	22	22.54	13
6	Germany	31	1948	44	62.84	16
7	Australia	30	1,268	22	42.27	15
8	France	27	1,458	43	54.00	13
9	Spain	26	800	16	30.77	10
10	Iran	21	470	7	22.38	9

The United States ranks second with 124 articles, accounting for 13.14% of the total. Its H-index is 30, and its publications have received 5,838 citations, with an average of 47.08 citations per article. Although the United States has fewer publications and a lower H-index compared to China, its higher average citation count reflects the broader recognition and impact of its research. Among the top ten countries, Germany stands out with 27 articles and the highest average citation count (62.84 per article), indicating the high quality and recognition of its research in this field.

Since 2017, China’s research output has grown consistently, particularly after 2020, when its annual publications accounted for more than half of the total in this field. Both China and the United States serve as key nodes connecting other countries, driving significant advancements and collaborations in the field (Figures 3D, E). Over the past 3 years, China has been the most active country in terms of research and publications, demonstrating strong support and keen interest from scholars in this area.

### 3.3 Authors and co-cited authors analysis

**FIGURE 4:**
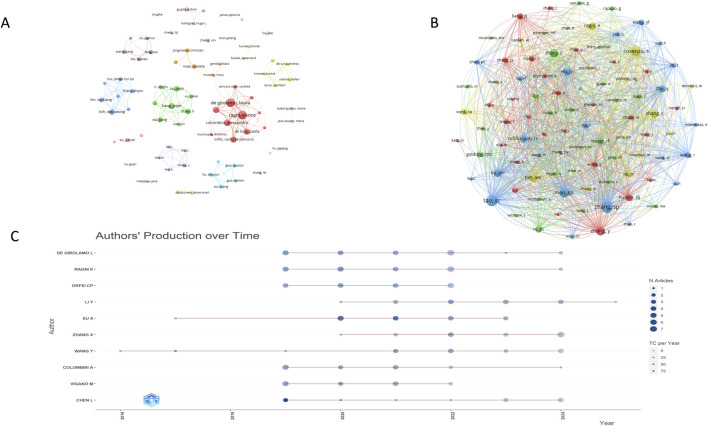
Analysis of author collaboration on extracellular vesicles and osteoarthritis. **(A)** Collaboration between authors based on VOSviewer; **(B)** Author co-citation coupling map; **(C)** Annual volumeof publications by top 10 authors.

A visual analysis of the authors included in the publications was conducted using VOSviewer software. When the publication threshold was set to one, 5,166 authors were identified as contributing to this research field; when the threshold was increased to five, 75 authors met this criterion. As shown in Figure 4A, the authors were grouped into distinct clusters based on their research areas and affiliated institutions.

The largest cluster comprises nine authors, primarily including Professor Laura de Girolamo and Professor Alessandra Colombini from Italy. The second-largest cluster consists of Professor Li Duan, Professor Li Xingfu, Professor Liang Yujie, Professor Wang Daping, Professor Xia Jiang, Professor Xiao Yin, Professor Xu Limei, and Professor Xu Xiao from Shenzhen University in China. Notably, these authors exhibit strong collaborative ties, making significant contributions to the advancement of the field (Figure 4A). Based on publication counts, Professor Laura de Girolamo and Professor Enrico Ragni from IRCCS Istituto Ortopedico Galeazzi in Italy have the highest number of publications, with 23 each (Table 2). It is worth noting that the top five authors by publication volume are all from Italy, highlighting the high level of collaboration and productivity among Italian scholars in this field. Chinese scholars Professor Liang Yujie and Professor Li Duan rank sixth. Professor SP Zhang from Singapore and Professor Tao SC from China are the most co-cited authors, with 350 and 329 co-citations, respectively (Table 3). In Figure 4B, the node size represents the co-citation count, and the line thickness indicates the co-citation strength between cited references. This visualization underscores the high recognition of authors such as Zhang SP, Tao SC, Alessandra Colombini, and Marco Viganò, reflecting the quality and academic impact of their work.

**TABLE 2:** Top 10 authors in terms of the number of published papers.

Rank	Author	Country	Record	Citations	Average citations	Total link strength	H-index
1	De Girolamo, Laura	Italy	23	500	21.74	134	13
2	Ragni, Enrico	Italy	23	532	23.13	122	13
3	Colombini, Alessandra	Italy	15	457	30.47	89	12
4	Vigano, Marco	Italy	14	454	32.43	83	11
5	Orfei, Carlotta Perucca	Italy	13	365	28.08	80	11
6	Liang, Yujie	China	13	769	59.15	78	9
7	Duan, Li	China	13	313	24.08	72	8
8	De Luca, Paola	Italy	12	736	61.33	74	9
9	Xu, Xiao	China	12	1,567	130.58	59	9
10	Toh, Wei Seong	Singapore	12	765	63.75	74	9

**TABLE 3:** Top 10 Co-cited authors in terms of the number of published papers.

Rank	Author	Country	Citations	Total link strength	H-index
1	Zhang, Sp	China	350	6,205	6
2	Tao, Sc	China	329	6,371	2
3	Cosenza, S	France	260	5,065	3
4	Mao, Gp	China	247	5,692	4
5	Zhang, Y	China	229	4,070	10
6	Toh, Ws	Singapore	225	3,595	1
7	Théry, C	France	217	3,533	1
8	Zhang, S	China	201	3,829	1
9	Wu, Jy	China	196	4,035	3
10	Liu, Yb	China	191	4,145	2

Among the top ten authors by publication volume, Professor Xu Xiao from Shenzhen University leads with an average of 130.58 citations per article, followed by Professor Toh Wei Seong and Professor De Luca Paola, with 63.75 and 61.33 citations per article, respectively. Figure 4C shows that most of these leading authors began their research around 2019, a year that marked a significant increase in the volume of publications in this field. This growth has continued through 2024, indicating that the field still holds substantial untapped potential.

### 3.4 Analysis of institution and research areas

**FIGURE 5:**
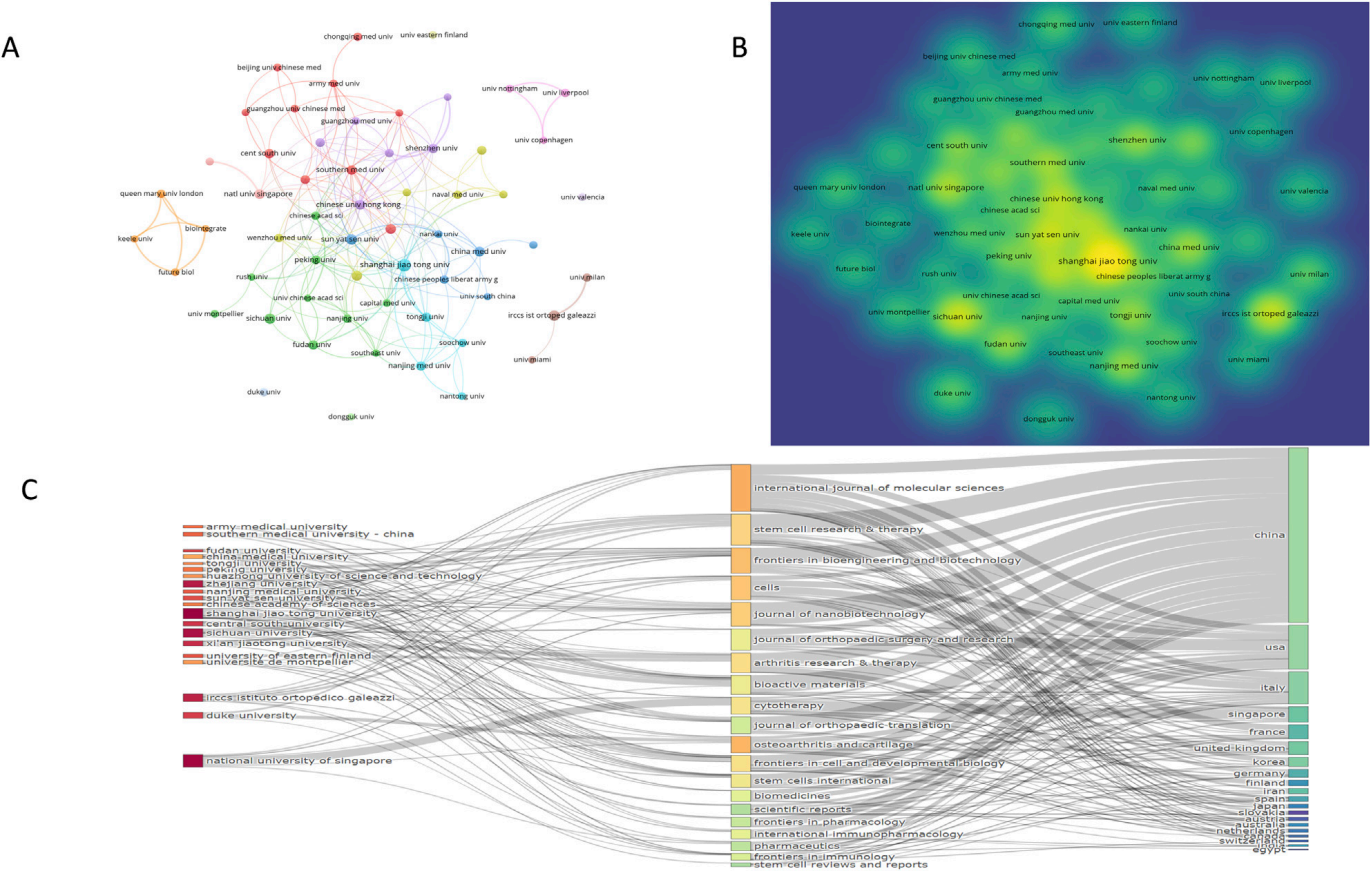
Analysis of institutional cooperation between extracellular vesicles and osteoarthritis **(A)** Collaboration between institution based on VOSviewer; **(B)** Hot spot distribution map of institutional issues; **(C)** Three plot map of institutions-journals-countries.

A visual analysis of institutions involved in the included literature was conducted using VOSviewer software. When the publication threshold was set at 1, 1,214 institutions were identified as active in this research field, while increasing the threshold to five reduced the number to 97 qualifying institutions. Figure 5A demonstrates distinct clustering patterns formed by authors from different research fields and institutions. These 76 institutions were organized into 5 clusters, with prominent representation from Chinese and American institutions in the larger clusters. Notably, institutions such as Zhejiang University, Sichuan University, Shanghai Jiao Tong University, and Shenzhen University demonstrated significant contributions (Figures 5A, B).

As shown in Table 4, Shanghai Jiao Tong University leads with 48 publications (5.08% of total publications), receiving 1,783 citations (37.15 citations per article). This is followed by Italy’s Sichuan University with 27 publications (2.86% of total), receiving 574 citations (21.26 per article), and subsequently IRCCS Istituto Ortopedico with 26 publications (2.75% of total) and 582 citations (22.38 per article). Both Figure 5C and Table 4 reveal that Chinese institutions occupy 8 of the top 10 positions by publication volume. This highlights China’s substantial engagement and contributions in this field, reflecting its growing recognition and institutional support within the research community.

**TABLE 4:** Top 10 Institutional in terms of the number of published papers.

Rank	Institutiona	Country	Record	Citations	Average citations	Total link strength
1	Shanghai Jiao Tong University	China	48	1783	Cosenza et al. (2017)	36
2	Sichuan University	China	27	574	21.26	5
3	IRCCS Istituto Ortopedico Galeazzi	Italy	26	582	22.38	9
4	Sun Yat-sen University	China	23	1,113	48.39	22
5	Zhejiang University	China	22	1,197	54.41	13
6	Chinese University of Hong Kong	China	21	1759	83.76	42
7	National University of Singapore	Singapore	21	2,322	110.57	14
8	China Medical University	China	19	739	38.89	11
9	Huazhong University of Science and Technology	China	19	288	15.16	7
10	Fudan University	China	18	381	21.17	14

### 3.5 Analysis of journals and research areas

**FIGURE 6:**
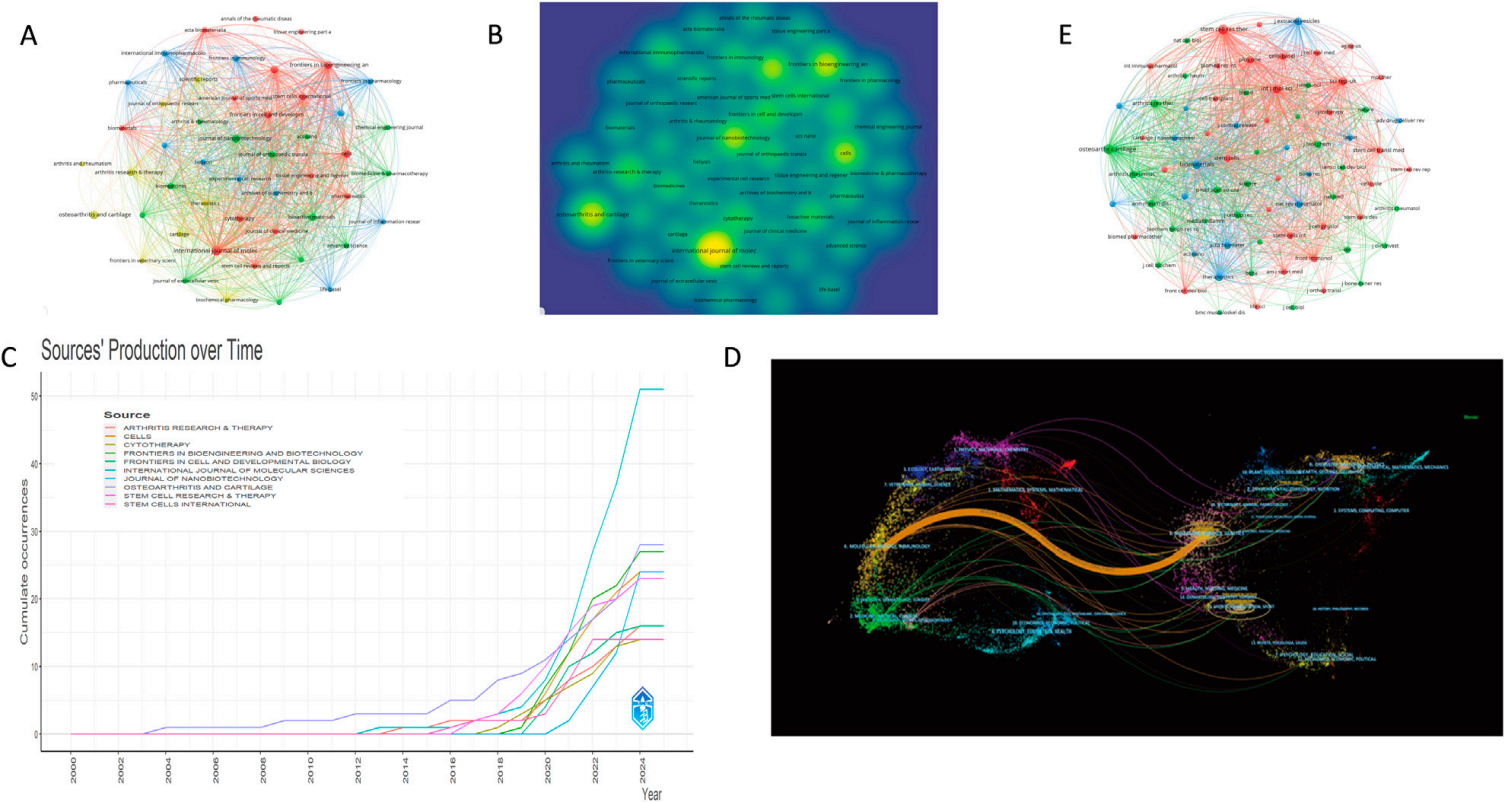
Journal collaboration analysis of extracellular vesicles and osteoarthritis. **(A)** Collaboration between journal based on VOSviewer. **(B)** Hot spot distribution map of journal issues; **(C)** Top 10 journals in terms of annual number of publications. **(D)** The dual-map overlay of journals contributed to publications. **(E)** Journal co-citation collaboration network mapping.

A visual analysis of journals in this field from 2000 to 2024 was conducted using VOSviewer software. When the publication threshold was set to one, 315 journals were identified as contributing to this research area; when the threshold was increased to five, 49 journals met this criterion. Figures 6A, B reveal that *Frontiers in Bioengineering and Biotechnology*, *International Journal of Molecular Sciences*, *Cells*, and *Stem Cell Research* & *Therapy* form the largest clusters, indicating their prominent role and high activity in publishing articles within this field. As shown in Table 5, the *International Journal of Molecular Sciences* leads with 51 articles, accumulating 757 citations and an average of 14.84 citations per article, alongside a 2024 impact factor of 3.7. Notably, the *Stem Cell Research & Therapy* published 23 articles, which received 2,050 citations with an average of 89.13 citations per article, demonstrating the highest average citation rate in this research domain. Figure 6C illustrates that publication numbers were relatively low between 2000 and 2014, but a marked increase began in 2019 and continued to rise through 2024. Most top-ten journals reached their peak in 2022 followed by a slight decline, with 2020–2024 representing the most prolific period.

**TABLE 5:** Top 10 Journal in terms of the number of published papers.

Rank	Journal	Country	Record	Citations	Average citations	H-index	Impact factor (2024)
1	International Journal of Molecular Sciences	Switzerland	51	757	14.84	11	3.7
2	Osteoarthritis and Cartilage	United Kingdom	28	742	26.50	14	5.1
3	Frontiers in Bioengineering and Biotechnology	Switzerland	27	680	25.19	11	4.5
4	Cells	Switzerland	24	412	17.17	16	5.3
5	Journal of Nanobiotechnology	United Kingdom	24	587	24.46	9	7.4
6	Stem Cell Research & Therapy	United Kingdom	23	2050	89.13	8	6.5
7	Arthritis Research & Therapy	United Kingdom	16	708	44.25	9	4.5
8	Frontiers in Cell and Developmental Biology	Switzerland	16	414	25.88	8	4.3
9	Cytotherapy	United Kingdom	14	93	6.64	9	4.4
10	Stem Cells International	United States of America	14	287	20.50	6	3.7

As shown in Figure 6D, the most frequently co-cited journals in bibliographic coupling analysis include *International Journal of Molecular Sciences*, *Stem Cell Research* & *Therapy*, *Biomaterials*, and *Osteoarthritis and Cartilage*. These journals have exerted significant influence on research and publications in this field, not only in terms of publication volume and co-citation frequency but also as critical channels for advancing scholarly discourse. Figure 6E presents a dual-map overlay of articles published between 2000 and 2024. Citation relationships are represented by colored lines on the right side (citing journals) and cited journals on the left. The analysis reveals a concentration of research in publications related to physics, materials science, chemistry, immunology, molecular biology, medicine, and clinical studies. Most cited journals originate from disciplines such as sports science, rehabilitation, materials science, chemistry, genetics, molecular biology, and physics. The interdisciplinary networks and collaborations reflect current developments and emerging areas of interest across these domains.

### 3.6 Analysis of references

**FIGURE 7:**
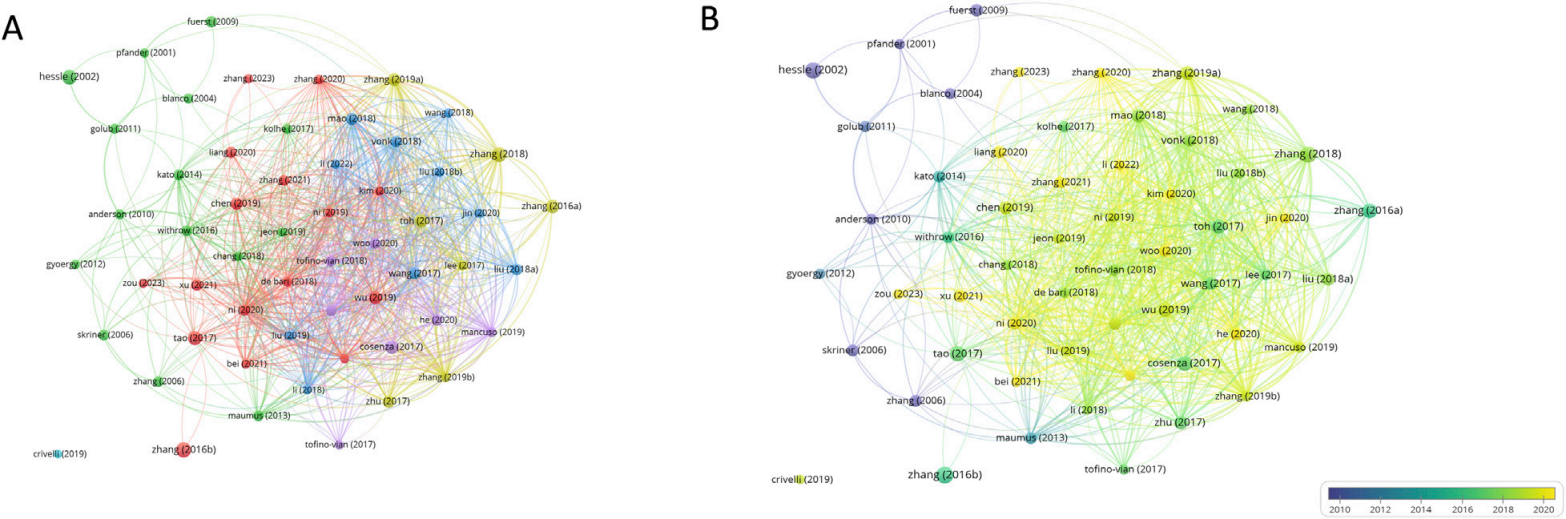
**(A, B)** Collaboration between publications based on VOSviewer.

As shown in Figures 7A, B, the studies were collectively analyzed based on their citations. Using VOSviewer, publications with ≥100 citations were analyzed, revealing the top-cited works. The leading study, *“Implant-Derived Magnesium Induces Local Neuronal CGRP Production to Promote Fracture Healing in Rats”* (Zhang Y. et al., 2016). Published in *Nature Medicine*, has accumulated 622 citations (an average of 77.75 citations per year). This groundbreaking research demonstrates that magnesium ions enhance calcitonin gene-related peptide (CGRP)-mediated osteogenic differentiation by activating the MAGT1-dependent transient receptor potential cation channel, Table 6.

**TABLE 6:** Top 10 cited publications ranked in the field.

Rank	Author	years	Average citations	Total link strength	Journal	JCR (2022)	Title	Details
1	Zhang Y. et al. (2016)	2016	622	0	nature medicine	Q1	Implant-derived magnesium induces local neuronal production of CGRP to improve bone-fracture healing in rats	Magnesium transporter protein 1 (MAGT1)-dependent and transient receptor potential cation channels are induced by elevated magnesium, suggesting that magnesium plays a role in CGRP-mediated osteogenic differentiation
2	Zhang et al. (2018)	2018	568	175	Biomaterials	Q1	MSC exosomes mediate cartilage repair by enhancing proliferation, attenuating apoptosis and modulating immune reactivity	By activating the AKT and ERK signalling pathways, exosomal CD73 facilitates cell proliferation and infiltration in cartilage repair. In contrast, inhibiting these signalling pathways decreases cell proliferation and migration without impacting matrix production
3	Tao et al. (2017)	2017	475	212	Theranostics	Q1	Exosomes derived from miR-140-5p-overexpressing human synovial mesenchymal stem cells enhance cartilage tissue regeneration and prevent osteoarthritis of the knee in a rat model	While SMSC-140-Exos effectively prevented OA in a rat model *in vivo*, it improved the migration and proliferation of ACs *in vitro* without affecting ECM secretion
4	Zhang S. et al. (2016)	2016	454	156	osteoarthritis and cartilage	Q1	exosomes derived from human embryonic mesenchymal stem cells promote osteochondral regeneration	The first evidence of human embryonic MSC exosomes’ efficacy in cartilage regeneration and their availability as a cell-free, off-the-shelf therapeutic alternative
5	Stefancin and Parker (2007)	2017	404	180	Scientific Reports	Q2	Mesenchymal stem cells derived exosomes and microparticles protect cartilage and bone from degradation in osteoarthritis	Exosomes and microvesicles/microparticles both prevent mice from developing OA *in vivo* and exhibit comparable chondroprotective and anti-inflammatory properties *in vitro*
6	Toh et al. (2017)	2017	321	123	Seminars in Cell & Developmental Biology	Q1	MSC exosome as a cell-free MSC therapy for cartilage regeneration: Implications for osteoarthritis treatment	This study offers fresh insights into the development of off-the-shelf, cell-free MSC therapeutics. It addresses the potential mechanisms of action of MSC exosomes in cartilage regeneration within the framework of their immunomodulatory and regenerative potential
7	Zhang et al. (2019)	2019	318	127	Biomaterials	Q1	MSC exosomes alleviate temporomandibular joint osteoarthritis by attenuating inflammation and restoring matrix homeostasis	MSC exosomes reduce IL-1β-induced nitric oxide and MMP13 production and increase s-GAG synthesis that IL-1β blocks
8	Wu et al. (2019)	2019	318	157	Biomaterials	Q1	miR-100-5p-abundant exosomes derived from infrapatellar fat pad MSCs protect articular cartilage and ameliorate gait abnormalities *via* inhibition of mTOR in osteoarthritis	Exosomes derived from infrapatellar fat pad (IPFP) MSCs regulate the mTOR-autophagy pathway through miR100-5p, preserving cartilage homeostasis and shielding articular cartilage from harm
9	Mao et al. (2018)	2018	288	144	Stem Cell Research & Therapy	Q1	Exosomes derived from miR-92a-3p-overexpressing human mesenchymal stem cells enhance chondrogenesis and suppress cartilage degradation *via* targeting WNT5A	Exosomal miR-92a-3p targets WNT5A to regulate cartilage growth and homeostasis; exosomal miR-92a-3p may function as a Wnt inhibitor
10	Zhu et al. (2017)	2017	283	125	Stem Cell Research & Therapy	Q1	Comparison of exosomes secreted by induced pluripotent stem cell-derived mesenchymal stem cells and synovial membrane-derived mesenchymal stem cells for the treatment of osteoarthritis	In mice OA models, injections of both iMSC-Exos and SMMSC-Exos reduce OA; however, iMSC-Exos has more therapeutic efficacy than SMMSC-Exos

Ranking second is *“Mesenchymal Stem Cell-Derived Exosomes Mediate Cartilage Repair by Enhancing Proliferation, Inhibiting Apoptosis, and Modulating Immune Responses”* (Zhang et al., 2018), with 568 citations (an average of 94.67 citations per year). This study highlights the molecular mechanism by which exosomal CD73 promotes cell proliferation and infiltration during cartilage regeneration through the activation of the AKT and ERK signaling pathways. Notably, it was the first to reveal that inhibiting this pathway does not affect matrix synthesis but significantly reduces cell migration and proliferation. In third place is *“Exosomes Derived from miR-140-5p-Overexpressing Synovial Mesenchymal Stem Cells Enhance Cartilage Regeneration and Prevent Osteoarthritis in Rat Knees”* (Tao et al., 2017), published in *Theranostics*, with 475 citations (an average of 67.86 citations per year). This study systematically demonstrates that SMSC-140-Exos simultaneously promote the migration, proliferation, and extracellular matrix (ECM) secretion of articular chondrocytes (ACs) *in vitro*, while also validating their therapeutic potential in preventing osteoarthritis (OA) in a rat model. The academic influence of these highly cited studies underscores their significant value in advancing the field.

### 3.7 Analysis of Keywords

**FIGURE 8:**
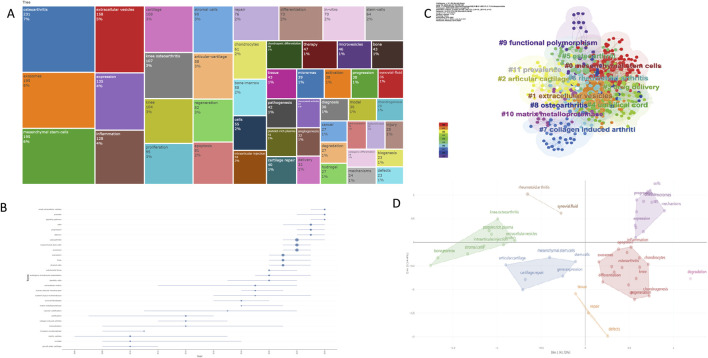
Keyword analysis of extracellular vesicles and osteoarthritis **(A)** Keyword Frequency Map **(B)** Keyword trend **(C)** and **(D)** Keywords clustering influencing factors analysis map and keyword clustering.

Keyword analysis is a critical tool for identifying the research focus of publications. Keywords appearing ≥100 times include *extracellular vesicle* (302 times), *mesenchymal stem cell* (253 times), *osteoarthritis* (179 times), *exosomes* (165 times), *expression* (113 times), *knee osteoarthritis* (106 times), and *inflammation* (104 times). Table 7 lists the top 20 keyword frequencies related to extracellular vesicles (EVs) in arthritis. In Figure 8A, larger squares represent higher keyword frequencies, while smaller squares indicate lower frequencies.

**TABLE 7:** Top 10 keywords in the list by frequency.

Rank	Keywords	Frequency	Centrality
1	extracellular vesicle	302	0.03
2	mesenchymal stem cell	253	0.02
3	osteoarthriti	179	0.04
4	exosm	165	0.03
5	expression	113	0.12
6	knee osteoarthriti	106	0.03
7	inflammation	104	0.04
8	knee	95	0.04
9	proliferation	86	0.03
10	cartilage	85	0.09

These keywords were organized into 10 clusters: #0 mesenchymal stem cells, #1 extracellular vesicles, #2 articular cartilage, #3 drug delivery, #4 umbilical cord, #5 osteoarthritis, #6 rheumatoid arthritis, #7 collagen-induced arthritis, #8 functional polymorphism, and #9 matrix metalloproteinase. These clusters encapsulate the research hotspots and focal areas of EVs in arthritis, as illustrated in Figures 8B–D. The thematic grouping of keywords further enhances the clarity and organization of research priorities in the study of EVs in osteoarthritis (OA).

## 4 Discussion

### 4.1 General information

From 2000 to 2024, the period from 2016 to 2024 marked a phase of rapid development in this field, with a significant increase in the number of publications. During this dynamic decade, researchers from China and the United States made substantial contributions to the field. China ranked first with 518 publications, 15,077 citations, an average of 29.11 citations per publication, and an H-index of 52. The United States ranked second with 124 publications, 5,838 citations, an average of 47.08 citations per publication, and an H-index of 30. Italy ranked third with 73 publications, an H-index of 23, and 1,741 citations (averaging 23.85 citations per publication). These countries demonstrated strong collaborative relationships and collectively advanced the development of this research field.

Professors De Girolamo, Laura and Ragni, Enrico each published 23 articles, ranking first with H-indices of 13 and average citation rates of 21.74 and 37.15 per article, respectively. Between 2020 and 2022, the top ten authors exhibited significant publication output, averaging at least three articles per year (Figure 4). In the institutional analysis, Shanghai Jiao Tong University led with 48 publications, an average of 37.5 citations per article, and a total link strength of 36. Sichuan University ranked second with 27 publications, an average of 21.26 citations per article, and a total link strength of 5. IRCCS Istituto Ortopedico Galeazzi ranked third, with 26 publications, an average of 22.38 citations per article, and a total link strength of 9.

These institutions, including Zhejiang University and Shenzhen University (SZU), exhibit close collaboration and have driven significant advancements in the field (Figure 5). In the journal analysis, *Frontiers in Bioengineering and Biotechnology*, *International Journal of Molecular Sciences*, *Cells*, and *Stem Cell Research & Therapy* rank as the top four journals, each publishing over 20 articles, reflecting their strong engagement and high publication frequency in this domain. A distinct clustering phenomenon is observed among these journals, with the largest cluster highlighted in red in the visualization.

### 4.2 Hotspots and trends

Cluster analysis in keyword analysis effectively summarizes research hotspots and trends. Key terms such as #0 mesenchymal stem cells and #1 extracellular vesicles are pivotal in extracellular vesicle (EV) research. These EVs are categorized into non-plant-derived and plant-derived EVs based on their origins, reflecting the diversity and scope of research in this field (Lv et al., 2020; Qiu et al., 2023).

### 4.3 Relationship between non-plant-derived EVs and OA

**FIGURE 9:**
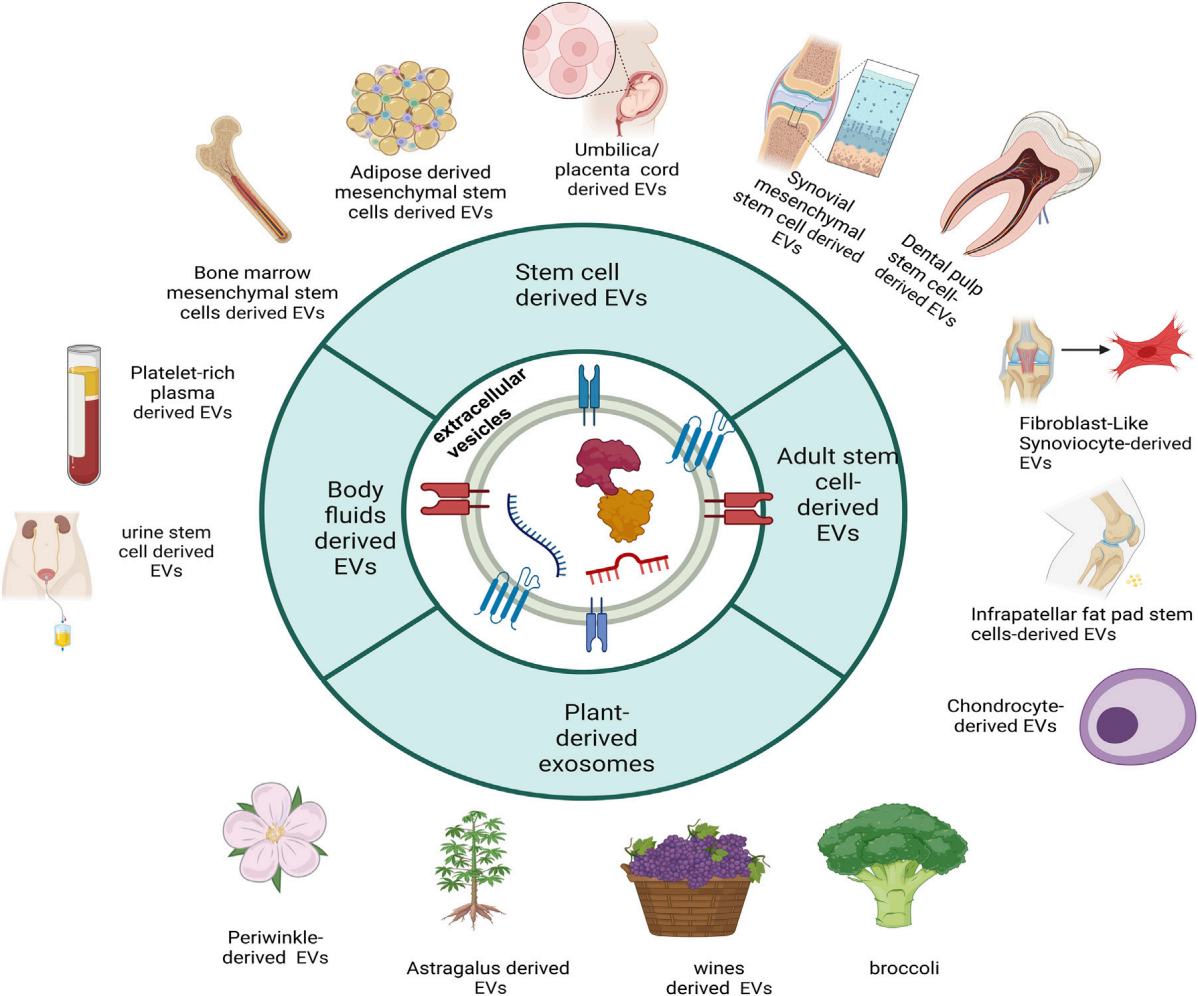
Evs from different sources play different roles in the treatment of OA (Chen et al., 2022; Yıldırım et al., 2024; Sarasati et al., 2023; Liu et al., 2024; Wang et al., 2021; Zhou et al., 2022; Sánchez et al., 2021; Meng et al., 2023; Kim et al., 2016; Gupta et al., 2021).

Non-plant-derived EVs and plant-derived EVs, have garnered significant research interest for their therapeutic potential in OA. Bone marrow mesenchymal stem cell (BMSC)-derived EVs are among the earliest and most extensively studied strategies, demonstrating efficacy in chondrocyte proliferation, anabolism, and apoptosis inhibition, Figure 9 (Pittenger et al., 1999).

Exosomes from BMSCs treated with decellularized extracellular matrix (dECM-BMSC-Exos) enhance cartilage repair *via* miR-3473b-mediated PTEN/AKT pathway activation (Zhang B. et al., 2023). In monosodium iodoacetate (MIA)-induced OA models, BMSC-derived exosomes upregulate collagen II and MMP13 expression (He et al., 2020), while TUC339-enriched exosomes promote macrophage polarization to the anti-inflammatory M2 phenotype, mitigating joint injury (Shen et al., 2023). BMSCs further regulate immunomodulation through autotaxin-YAP pathway modulation (Wang et al., 2021). Dental pulp stem cells (DPSCs), sharing surface marker similarities with BMSCs but exhibiting superior proliferative capacity, enhance chondrocyte repair *via* intra-articular exosome delivery. These exosomes suppress TRPV4-mediated osteoclast activity, reducing subchondral bone remodeling and cartilage degradation in murine OA models (Fu et al., 2023). Adipose-derived stem cells (ADSCs), prized for their abundance and accessibility, secrete exosomes (ADSC-Exos) that promote cartilage regeneration and reduce inflammation. Elastogen (TE) pretreatment amplifies ADSC-Exo secretion and upregulates miR-451-5p, enhancing chondrocyte matrix synthesis and repair in anterior cruciate ligament transection (ACLT) models (Meng et al., 2023).

Clinical translation efforts include chemically defined medium (CDM)-cultured small EVs (CDM4-sEVs), which exhibit high purity and stimulate chondrocyte proliferation, migration, and differentiation. CDM4-sEVs inhibit osteochondral degeneration *in vivo*, underscoring their therapeutic potential (Hanai et al., 2023). Synovial MSC-derived exosomes (SMSC-Exos) drive chondrocyte migration and collagen synthesis *via* Wnt5a/5b-YAP signaling, albeit at the expense of SOX9-dependent extracellular matrix production (Park et al., 2015; Liu and Lefebvre, 2015). SMSC-Exos also mitigate IL-1β-induced cartilage degradation through NRP1 targeting and miR-485-3p-mediated PI3K/Akt suppression (Qiu et al., 2024). Umbilical cord-derived MSC exosomes (hucMSC-EVs), aligned with keyword cluster #4 (“umbilical cord”), demonstrate chondroprotective effects by enhancing COL2A1 and aggrecan expression while suppressing ADAMTS5 and MMP13. METTL3-mediated NLRP3 m6A methylation reduction underlies their anti-inflammatory action (Zhou et al., 2022; Jin et al., 2021).

Exosomes that overexpress miR-92a-3p in MSCs promote matrix gene expression and cartilage growth. On the other hand, exosomes that block miR-92a-3p decrease chondrogenic differentiation and upregulate WNT5A expression, which reduces the formation of cartilage matrix. WNT5A is the direct target of miR-92a-3p, which inhibits its activity. Exosomes overexpressing miR-92a-3p could potentially serve as inhibitors of the Wnt signalling pathway and may be developed as therapeutic agents to modify the disease process in OA (Mao et al., 2018). However, more investigation is required to clarify the underlying mechanisms in more depth. Perinatal stem cells, including embryonic MSCs (EMSCs) and amniotic membrane MSCs (hAMSCs), balance extracellular matrix synthesis and degradation. EMSC-EVs preserve chondrocyte phenotype under inflammatory conditions (Wang et al., 2017), while hAMSCs outperform ADSCs in modulating synovial macrophage polarization and glycosaminoglycan preservation (Topoluk et al., 2018).

**FIGURE 10:**
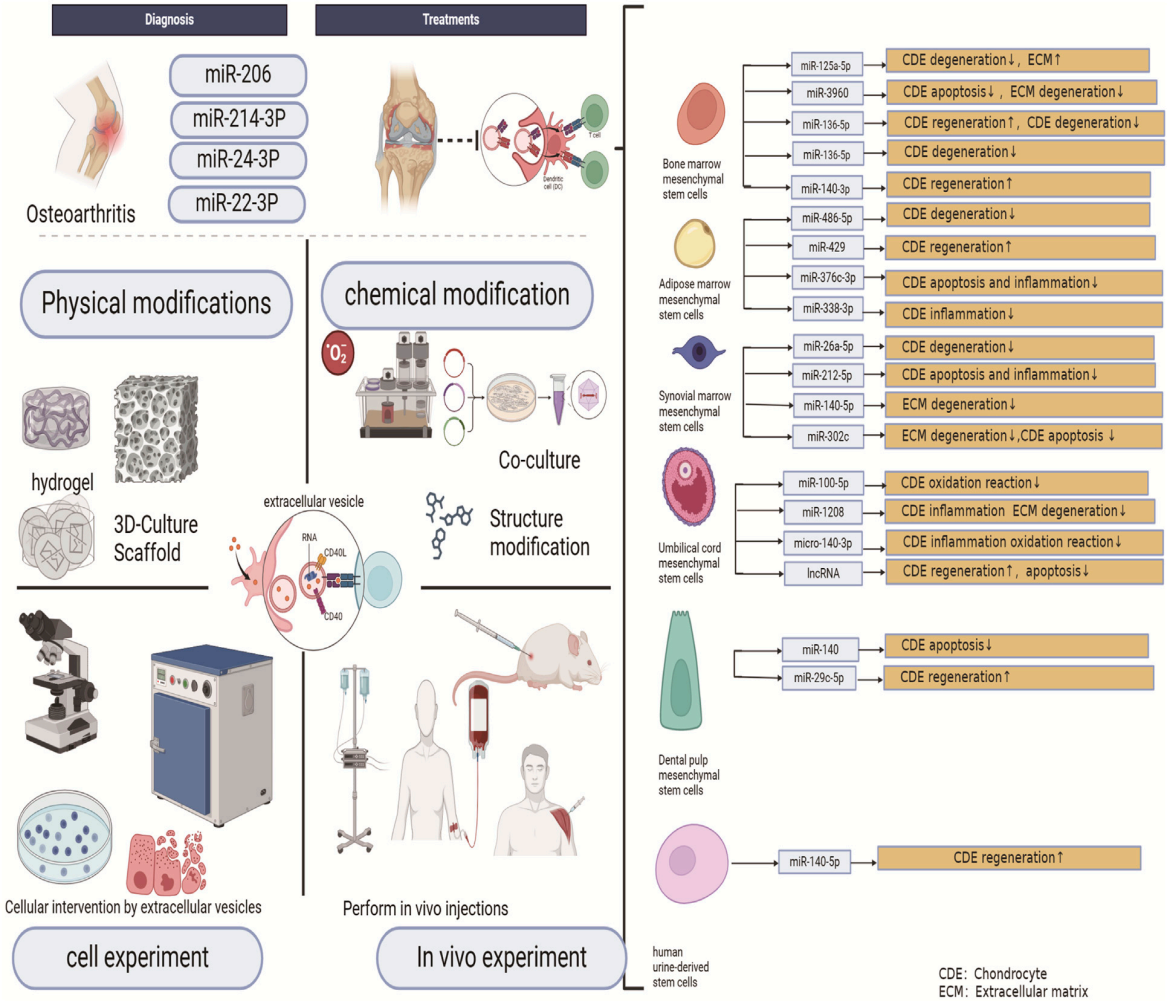
Role of different sources of evs and carrying miRNAs in diagnosis and treatment in OA (Gupta et al., 2021; Sánchez et al., 2021; Wan et al., 2023; Wang et al., 2021; Xu et al., 2023; Zhang et al., 2020; Zhou et al., 2022; Zhou et al., 2020; Zou et al., 2023).

Amniotic fluid stem cell exosomes (AFSC-Exos) deliver immunomodulatory factors (e.g., TGF, HGF) to attenuate inflammation and fibrosis (Beretti et al., 2018). hUSC-140-Exos further promote cartilage regeneration *via* VEGFA signaling (Liu et al., 2022). Collectively, these findings highlight MSC-EVs as versatile tools for OA intervention, though deeper mechanistic insights are needed to optimize clinical applications (Figures 9, 10) (Cheng et al., 2023; Yu et al., 2022; Kim et al., 2020; Wang S. et al., 2022).

### 4.4 Relationship between plant-derived EVs and OA

Plant extracellular vesicles (PELNs), secreted by most plant cells, share compositional similarities with animal-derived exosomes but exhibit distinct molecular profiles influenced by plant species, environmental factors, and isolation methods. These vesicles carry diverse bioactive molecules, including proteins, lipids, and nucleic acids, with demonstrated therapeutic potential in OA. Notably, Yıldırım et al. (Yıldırım et al., 2024) reported that tomato-derived EVs significantly upregulated chondrogenic markers—aggrecan, SRY-box transcription factor 9 (SOX9), and cartilage oligomeric matrix protein (COMP)—in human chondrocytes, thereby enhancing cartilage regeneration. Mechanistically, T-EVs facilitated growth factor delivery to chondrocytes, creating a pro-regenerative microenvironment that supports neo-cartilage formation and maturation.

Complementing these findings, Chen et al. (2022) engineered spinach-derived EVs functionalized with chondrocyte membrane fragments. Upon light exposure, these hybrid vesicles elevated intracellular adenosine triphosphate (ATP) and reduced nicotinamide adenine dinucleotide phosphate (NADPH) levels in degenerated chondrocytes. This metabolic reprogramming enhanced anabolic activity, restored cartilage homeostasis, and attenuated OA progression in a murine model. Together, these studies highlight the species-specific bioactivity of plant-derived EVs and their potential as tunable nanotherapeutics for OA intervention (Figure 9) (Chen et al., 2022; Yıldırım et al., 2024; Sarasati et al., 2023; Liu et al., 2024; Wang et al., 2021; Zhou et al., 2022; Sánchez et al., 2021; Meng et al., 2023; Kim et al., 2016; Gupta et al., 2021).

### 4.5 Relationship between EVs of body fluid origin and OA

Exosomes generated by somatic sources, like platelet-rich plasma-derived exosomes (PRP-Exos) (Zhang et al., 2022), are also very important. PRP-Exos decreases apoptosis, encourages chondrocyte migration and proliferation, and blocks the release of the inflammatory cytokine TNF-α. They mitigate the advancement of OA by reversing the effects of IL-1β on essential protein expression in the Wnt/β-catenin signalling pathway (Liu et al., 2019). It found that SDF-1 in PRP-Exo mediates the migration of bone marrow mesenchymal stem cells (mBMSCs) to the injury site *via* the CXCR4 receptor. Concurrently, TGF-β1 activates the Smad2/3 pathway, promoting their differentiation into chondrocytes by inducing Smad2/3 phosphorylation, upregulating the expression of SOX9 and COL II, and driving chondrogenic differentiation of mBMSCs. PRP-Exo inhibits IL-1β-induced phosphorylation of p65 (a subunit of NF-κB) and STAT3, thereby reducing the expression of MMP13 and COL X, which suppresses cartilage matrix degradation and chondrocyte hypertrophy. Additionally, PDGF-BB and TGF-β1 block the pro-inflammatory cytokine cascade by inhibiting IKKα and STAT signaling pathways (Figures 9, 10) (Zhang et al., 2022).

### 4.6 Relationship with OA after chemical modification of EVs

Another approach to enhance the functionality of non-plant-derived EVs involves modifying donor cells to improve their biochemical properties, thereby increasing their clinical applicability. One method includes genetic modification, where stem cells or EVs are engineered to overexpress specific miRNAs, circRNAs, or lncRNAs to achieve targeted effects (Huang et al., 2022; Wang et al., 2023). Likewise, lncRNA MEG-3-modified BMSCs-EVs slow down the advancement of OA by reducing IL-1β-induced chondrocyte senescence and apoptosis (Jin et al., 2021). Furthermore, it was discovered that TNF-α-induced exosomes improved HUVEC cell motility, invasion, and angiogenesis *via* the miR-200a-3p/KLF6/VEGFA axis (Zhang et al., 2023c). Another method to modify donor cells involves cell co-culture. Curcumin, the primary biological component of turmeric, has been utilized to treat MSCs, resulting in exosomes that reduce DNA methylation in the promoter regions of miR-143 and miR-124, thereby increasing their expression. Additionally, binding sites for miR-143 and miR-124 are found in the 3′untranslated region (3′UTR) of NF-kB and ROCK1, respectively, indicating that these miRNAs can directly target NF-kB and ROCK1. As a result, exosomal therapy considerably slows the advancement of OA 70 by restoring normal NF-kB and ROCK1 expression (Qiu et al., 2020). Controlling the concentration of oxygen is another strategy. It has been demonstrated that hypoxia preserves the characteristics of stem cell development, impacting their phenotypic and function and boosting the therapeutic potential of stem cells and the EVs they produce. EVs produced from umbilical cord stem cells (USC-EVs) under hypoxic settings were found to be far more effective in promoting chondrocyte migration and proliferation than EVs produced under normoxic conditions. The enhancement was made possible by using USC-EVs to transfer miR-26a-5p to chondrocytes (Wan et al., 2022), Figure 10 (Gupta et al., 2021; Sánchez et al., 2021; Wan et al., 2023; Wang et al., 2021; Xu et al., 2023; Zhang et al., 2020; Zhou et al., 2022; Zhou et al., 2020; Zou et al., 2023).

### 4.7 Relationship with OA after physical modification of EVs

Physical interventions to engineer EVs offer innovative strategies for OA treatment, encompassing 3D culture systems, biomaterial encapsulation, and targeted drug delivery. Compared to conventional 2D methods, 3D culture techniques—such as spinner flasks, hanging droplets, and pellet systems—significantly enhance MSC exocytosis efficiency, amplifying paracrine therapeutic effects (Lee and Lee, 2022). Chen et al. (2019) developed a 3D-printed scaffold integrating cartilage extracellular matrix (ECM), gelatin methacrylate (GelMA), and exosomes, which facilitated cartilage regeneration by promoting chondrocyte migration and polarizing synovial macrophages toward the anti-inflammatory M2 phenotype.

Hydrogel-based EV delivery outperforms other biomaterials, enhancing bone marrow MSC (BMSC) migration, proliferation, and differentiation to accelerate cartilage repair and ECM remodeling. MSC-derived nano-vacuoles (MSC-NVs) encapsulated in hydrogels exhibit superior mechanical stability and biocompatibility. In murine OA models, hydrogel-loaded MSC-NVs improved matrix synthesis, reduced catabolic factor secretion, and attenuated disease severity, while GelMA-NVs suppressed inflammation *via* M2 macrophage polarization (Zhang et al., 2021; Pang et al., 2023). Wan et al. (2023) advanced this approach with a photocrosslinkable spherical GelMA hydrogel encapsulating cartilage-targeting exosomes (W-Exo@GelMA), which enhanced joint retention and chondrocyte specificity, effectively delaying OA progression through dual anabolic promotion and catabolic inhibition.

Integrating hydrogels with 3D-printed scaffolds provides tailored mechanical support and joint-mimetic microenvironments, optimizing EV release kinetics and joint homeostasis (Vijayavenkataraman et al., 2018; Sun et al., 2023). Li et al. (2023) engineered a biomimetic double-network hydrogel scaffold incorporating adipose MSC-derived exosomes and decellularized ECM, which enhanced BMSC adhesion, differentiation, and osteochondral regeneration in rat OA models. Rat BMSCs displayed improved adhesion, spreading, migration, proliferation, and chondrogenic and osteogenic differentiation *in vitro* with the help of this scaffold. It successfully promoted cartilage and subchondral bone tissue regeneration in a rat model of OA. EVs serve as versatile carriers for diverse therapeutics, including antisense oligonucleotides (Yang et al., 2021), mRNA (Bu et al., 2021), siRNA (Huang et al., 2021), protein/peptide drugs (Yang et al., 2018; Wu et al., 2023), and curcumin (Sun et al., 2010). For instance, Xu et al. (2021) engineered exosomes to deliver kartogenin (KGN), boosting intracellular concentrations and chondrogenesis in synovial fluid MSCs, demonstrating efficacy *in vitro* and *in vivo*. These advancements underscore the potential of physically engineered EVs to revolutionize OA therapy through precision targeting, controlled release, and enhanced regenerative outcomes.

### 4.8 Relationship with arthritis after physical modification of EVs

EVs, particularly exosomes, have emerged as pivotal players in the pathophysiology, diagnosis, and treatment of rheumatoid arthritis (RA) and OA. Both conditions share common features, including cartilage degeneration, synovial inflammation, structural bone alterations, pain, and functional impairment. EVs, with their stability in circulation and minimally invasive sampling, offer a promising avenue for early disease detection and intervention, potentially improving patient outcomes (Liu et al., 2023).

In OA, reduced expression of miR-193b-3p in plasma exosomes correlates with inflammatory activity and joint degradation, positioning it as a potential biomarker for early disease detection and monitoring (Meng et al., 2018; Wang et al., 2012). Exosomal miRNAs, such as miR-let-7b, modulate inflammatory responses by targeting Toll-like receptors (TLRs) and promoting M1 macrophage polarization, leading to the secretion of pro-inflammatory cytokines like IL-1, IL-6, and TNF (Kim et al., 2016).

Conversely, MSC-derived EVs exhibit immunomodulatory effects, suppressing T cell proliferation, enhancing regulatory T cells (Tregs), and ameliorating inflammation in arthritic models (Cosenza et al., 2018). Despite their therapeutic potential, the precise mechanisms by which EVs influence OA progression—through inflammatory modulation, cellular senescence, and metabolic regulation—remain incompletely understood. The complexity of OA pathogenesis necessitates a multifaceted diagnostic and therapeutic approach, as reliance on single biomarkers is insufficient for comprehensive disease management. EVs represent a promising cell-free therapeutic platform, with significant potential for drug delivery and targeted therapy. However, critical questions regarding exosome sourcing, quality, dosage, and functional mechanisms require further exploration. Future research should focus on optimizing EV-based strategies to fully harness their diagnostic and therapeutic potential, ultimately advancing the management of RA and OA.

### 4.9 Advantages and limitations of research

In contrast to traditional literature reviews, bibliometric visualisation and analysis using software such as CiteSpace, VOSviewer, and the R package bibliometrics can effectively demonstrate the research hotspots and critical areas in the field, giving scholars valuable references and guiding future research directions more comprehensively.

However, this study has several limitations that should be acknowledged. Firstly, it relies solely on the Web of Science (WOS) core database, which may introduce biases and errors in understanding the overall trends and scope of publications. Secondly, due to the multidisciplinary nature of the field involving various research aspects, some publications related to EVs in the diagnosis and treatment of OA may not be fully captured, potentially limiting the scope of this study.

Furthermore, the study did not provide a complete assessment of the research focus and quality of each retrieved article. However, it provides novel possibilities for this field’s future study directions. It is advised that various databases should be integrated to progress in the future, and different analysis techniques should be used to investigate more extensive and in-depth study avenues. This approach could enhance our understanding of EVs’ role in OA diagnosis and treatment and contribute to the progression of research in this critical area.

## 5 Conclusion

This comprehensive bibliometric analysis evaluated global research trends on EVs in OA from 2000 onward, revealing sustained growth in annual publication rates. China emerged as the leading contributor, followed by the United States. Prof. Laura Girolamo ranked as the most prolific and cited author. The study systematically investigated EVs’ dual role in OA pathogenesis and therapy. Mechanistic insights highlighted their potential as early diagnostic biomarkers *via* high-expression factors and as pathogenic drivers *versus* regenerative agents influencing chondrocyte integrity. Furthermore, EVs demonstrated therapeutic modulation through cargo delivery and synergistic integration with biomaterials or genetic engineering, offering avenues to attenuate OA progression. This synthesis underscores EVs’ multifaceted impact on OA diagnostics and targeted intervention strategies.
